# A15 CX3CR1^+^ MYELOID CELL-INTRINSIC NOD2 SIGNALING LIMITS CROHN’S DISEASE-ASSOCIATED INTESTINAL FIBROSIS

**DOI:** 10.1093/jcag/gwaf042.015

**Published:** 2026-02-13

**Authors:** T Mukherjee, S Patel, M Narimatsu, J Yadav, D Tsang, G Bayer, M Kuypers, B K Tsankov, P H Shah, C Herbozo, D Trcka, T Mallevaey, A Ayyaz, G Bader, S Girardin, J Wrana, D J Philpott

**Affiliations:** University of Toronto Temerty Faculty of Medicine, Toronto, ON, Canada; Lunenfeld-Tanenbaum Research Institute, Toronto, ON, Canada; Lunenfeld-Tanenbaum Research Institute, Toronto, ON, Canada; University of Toronto Temerty Faculty of Medicine, Toronto, ON, Canada; University of Toronto Temerty Faculty of Medicine, Toronto, ON, Canada; University of Toronto Temerty Faculty of Medicine, Toronto, ON, Canada; University of Toronto Temerty Faculty of Medicine, Toronto, ON, Canada; University of Toronto Temerty Faculty of Medicine, Toronto, ON, Canada; University of Toronto Temerty Faculty of Medicine, Toronto, ON, Canada; University of Toronto Temerty Faculty of Medicine, Toronto, ON, Canada; Lunenfeld-Tanenbaum Research Institute, Toronto, ON, Canada; University of Toronto Temerty Faculty of Medicine, Toronto, ON, Canada; University of Calgary, Calgary, AB, Canada; University of Toronto Temerty Faculty of Medicine, Toronto, ON, Canada; University of Toronto Temerty Faculty of Medicine, Toronto, ON, Canada; Lunenfeld-Tanenbaum Research Institute, Toronto, ON, Canada; University of Toronto Temerty Faculty of Medicine, Toronto, ON, Canada

## Abstract

**Background:**

Crohn’s disease (CD) is a chronic inflammatory bowel disease that often progresses to fibrostenotic disease, requiring surgical intervention. Intestinal fibrosis (IF) is a debilitating outcome of chronic inflammation, excessive collagen deposition, abnormal cellular functions and defective tissue remodeling. CD stems from a complex interplay of host genetics, immune dysregulation, microbial imbalance, and environmental cues. Notably, loss-of-function variants in nucleotide-binding oligomerization domain-containing protein 2 (NOD2) – a cytosolic innate-immune receptor and the strongest genetic risk factor for CD – increase susceptibility to IF. Yet, the cell-intrinsic mechanisms by which NOD2 deficiency promotes IF remain unclear.

**Aims:**

(1) To identify the cellular and molecular mediators that predispose the host with the CD-associated risk gene, NOD2 to IF. (2) To investigate how NOD2 dysfunction mediates inter- and intra-cellular rewiring during IF?

**Methods:**

We employed a single-cell RNA-sequencing (scRNA-seq) approach to explore the cellular landscape in our CD-mouse model, where littermate wild-type (WT) and *Nod2*^-/-^ mice were challenged with a chronic inflammatory insult regime, using dextran sulfate sodium (cDSS) for 3 cycles. Complementary approaches such as quantitative real-time PCR (qRT-PCR), immunohistochemistry (IHC), RNA-*in situ* hybridization (RNA-ISH), flow cytometric analyses and conditional-knockout mice are used to validate cell populations and molecular signatures linked with IF.

**Results:**

Leveraging single-cell transcriptomics analysis, we identified the emergence of a unique cluster of *Pi16*^*+*^ progenitor cells, termed FAET (Fibroblast and Endothelial Transition) cells in the fibrotic gut. Our *in silico* cell-fate mapping analysis revealed FAET cells transdifferentiate into inflammation-associated fibroblasts (IAFs) instead of endothelial cells (EndoCs), causing loss of crypt microvasculature in *Nod2*^*-/-*^ mice. Cellular interactome analysis and genetic studies revealed that CX3CR1^+^ myeloid cell-intrinsic NOD2 signaling maintains CX3CR1^+^CD206^+^ gut resident macrophages, supporting an anatomical niche conducive to FAET-to-EndoCs transition over IAFs, thereby aiding wound healing. Consistently, ileal biopsies from CD patients with *NOD2* variants exhibit reduced CD206^+^ macrophages and disrupted pro-restitutive stromal-immune interaction networks.

**Conclusions:**

CX3CR1+ myeloid cell-intrinsic NOD2 signaling defines a pro-restitutive stromal-immune niche that limits intestinal fibrosis associated with CD. Our study offers new cellular and molecular insights into devising complementary and transformative therapeutic strategies to prevent IF in CD patients.

**Funding Agencies:**

CIHRThe American Association of Immunologists (AAI) Intersect Fellowship Program for Computational Scientists and Immunologists

